# Impression Disinfection and Its Effect on Dimensional Accuracy and Surface Detail in the Times of COVID-19: An In Vitro Study

**DOI:** 10.7759/cureus.55931

**Published:** 2024-03-11

**Authors:** Niharika Sabharwal, Aman Arora, Viram Upadhyaya, Monika M Sehgal, Karvika Nayak, Setu Katyal, Manisha Dahiya, Chandrama Pratap, Radhika Sharma

**Affiliations:** 1 Prosthodontics, Institute of Technology and Science (ITS) Dental College, Ghaziabad, IND; 2 Prosthodontics, JN Kapoor Dayanand Anglo Vedic (DAV) Dental College, Yamunanagar, IND; 3 Endodontics, Institute of Technology and Science (ITS) Dental College, Ghaziabad, IND

**Keywords:** surface detail reproduction, dimensional accuracy, herbal disinfectant, virucidal, ultraviolet radiation, ozonated water, peracetic acid, impression disinfection

## Abstract

Introduction

The disinfection of impressions is crucial to eliminate the viral and other microbial loads to prevent the cross contamination of diseases. The aim of this study was to compare the effect of different virucidal disinfecting methods on the dimensional accuracy and surface detail reproduction (SDR) of impression materials.

Methods

A total of 160 samples were fabricated with different impression materials using zinc oxide eugenol (Group 1), alginate (Group 2), polyether (Group 3), and addition silicone (Group 4) impression materials, each containing 40 samples (n=40). These groups were further divided into Subgroups A, B, C, and D (n=10) based on the disinfecting method used. Disinfection was carried out using 0.2% peracetic acid (A), a natural polymer of glucosamine (B), ultraviolet (UV) radiation (C), and ozonated water (D). The disinfected impressions were poured in type IV gypsum, and the obtained casts were checked for dimensional accuracy and surface detail reproduction (SDR). For dimensional accuracy, a one-way analysis of variance (ANOVA) test and, for surface detail reproduction, the chi-square test were used to compare the different subgroups of each impression material separately.

Results

Zinc oxide eugenol samples showed the lowest mean dimensional change when disinfected with 0.2% peracetic acid (1A=154.1 µm), and alginate showed the lowest mean dimensional change when disinfected using ozonated water (2D=134.9 µm). On the other hand, the lowest mean dimensional change observed in polyether and addition silicone samples was those which were disinfected using UV radiation (3C=100.9 µm and 4C=113.5 µm). Surface detail was reproduced adequately in most of the samples.

Conclusion

A 0.2% peracetic acid could be used to disinfect zinc oxide eugenol impressions, ozonated water for alginate impressions, and UV radiation for polyether and addition silicone impressions.

## Introduction

Since the World Health Organization (WHO) declared the COVID-19 outbreak as a pandemic on 11 March 2020, medical research has gained unprecedented momentum due to the lethality of SARS-CoV-2 and its global spread in just a few months. While vaccines play a key role in preventing viral epidemics and pandemics, once an outbreak has occurred, the implementation of disinfection measures to limit spread becomes paramount. Dental healthcare professionals, which include dentists and dental auxiliaries, are at a high risk of contracting diseases that spread through air, water droplets, and fomites.

One of the important steps in treating dental cases is making a dental impression on the patient. Dental impressions can act as vehicles for various types of viruses, e.g., hepatitis B and C, HIV, herpes simplex, Ebola, Middle East respiratory syndrome-related coronavirus (MERS-CoV), and, now, SARS-CoV-2 [1].

According to the American Dental Association (ADA), the dental impression should be immediately disinfected after retrieval from the mouth [2]. Until 1991, the recommended procedure for the disinfection of impressions was rinsing under running water with which only 40% of bacteria, viruses, and fungi were removed [3]. In recent times, a pre-wash of the impression with running water is advocated first to cast off all particles, blood, and saliva prior to the active disinfection procedure [4].

The currently used chemical disinfectants such as glutaraldehyde, sodium hypochlorite, and iodophors are known to be corrosive, potentially harmful chemicals [5], not compatible with all types of impression materials, and have been known to affect the properties of impression materials. In recent years, new chemical and physical disinfecting methods with virucidal action have been introduced. The new methods include chemical disinfectants such as peracetic acid and a natural polymer of glucosamine and physical disinfecting methods employing ozonated water and ultraviolet (UV) radiation, which were used in this study. They are biocompatible and are safe for the regular disinfection of impression materials. Since they produce nontoxic by-products or none at all in the case of ultraviolet radiation and ozonated water, the distortion of the impression materials becomes potentially low.

The role of a disinfectant should, ideally, be of a dual purpose. It must be an effective antimicrobial agent, preferably antiviral, yet cause no adverse response to the dimensional accuracy and surface detail reproducibility of the impression material and the resultant gypsum cast [6]. Accuracy is crucial for the optimal functioning of a prosthesis. Hence, studying the effect of disinfectants on the dimensional accuracy and surface detail reproducibility of dental impression materials is important.

The objective of the proposed study was to understand and compare the effect of peracetic acid, a natural polymer of glucosamine, ultraviolet radiation, and ozonated water as new, safer, economical, and effective virucidal disinfecting methods on the commonly used zinc oxide eugenol, alginate, addition silicone, and polyether impression materials and to recommend a suitable virucidal disinfecting method for these impression materials based on their effect on dimensional accuracy and surface detail reproduction (SDR).

## Materials and methods

The study was conducted at the Department of Prosthodontics of JN Kapoor DAV Dental College, Yamunanagar, Haryana, India, from January 2023 to April 2023. The impression materials used in this study were zinc oxide eugenol (Coltene, Cuyahoga Falls, OH), alginate (Impreceed, GC, Tokyo, Japan), polyether (Impregum Soft, 3M ESPE, Saint Paul, MN), and addition silicone (Ad-Sil Acura, Prime Dental, Thane, India). The virucidal disinfectants used in the study included 0.2% peracetic acid (Chemtex, Bangalore, India), a natural polymer of glucosamine (Ecosan, Medizysis, Delhi, India), UV radiation (UVC lamp, Philips, Kolkata, India), and ozonated water (ozone generator, Prestige, Loro Ciuffenna, Italy). The casts were poured from type IV gypsum (Kalrock, Kalabhai Karson Pvt Ltd, Mumbai, India).

**Figure 1:**
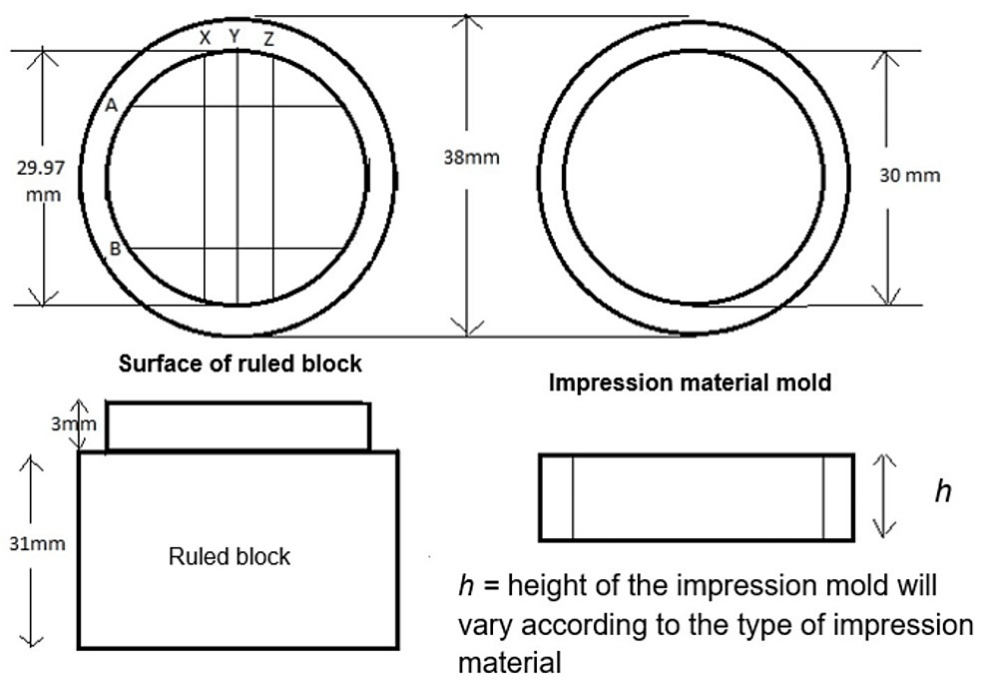
Components of the master model

A master model was designed and fabricated according to ADA specification numbers 18 and 19 [7,8]. It comprised a ruled block, a riser, a perforated disc, and a gypsum casting mold (Figure 1). Two risers of heights 5 mm and 7 mm, which served as impression material molds, were designed. The 5 mm riser was used with zinc oxide eugenol and polyether impression materials, whereas the 7 mm riser was used with alginate and addition silicone impression materials. A perforated disc that would serve to apply pressure over the setting impression material was designed having a diameter of 38 mm.

Forty samples each of zinc oxide eugenol (Group 1), alginate (Group 2), polyether (Group 3), and addition silicone (Group 4) were made using the master model. This was succeeded by the retrieval of the samples in conjunction with the riser and perforated disc assembly. The impressions were inspected with the naked eye for voids and, if present, were discarded.

The groups were further divided into subgroups (n=10) depending on the disinfecting method used. In Subgroup A, impression samples were disinfected with 0.2% peracetic acid (1A, 2A, 3A, and 4A), in Subgroup B with a natural polymer of glucosamine (1B, 2B, 3B, and 4B), in Subgroup C with UV radiation (1C, 2C, 3C, and 4C), and in Subgroup D with ozonated water (1D, 2D, 3D, and 4D).

**Figure 2:**
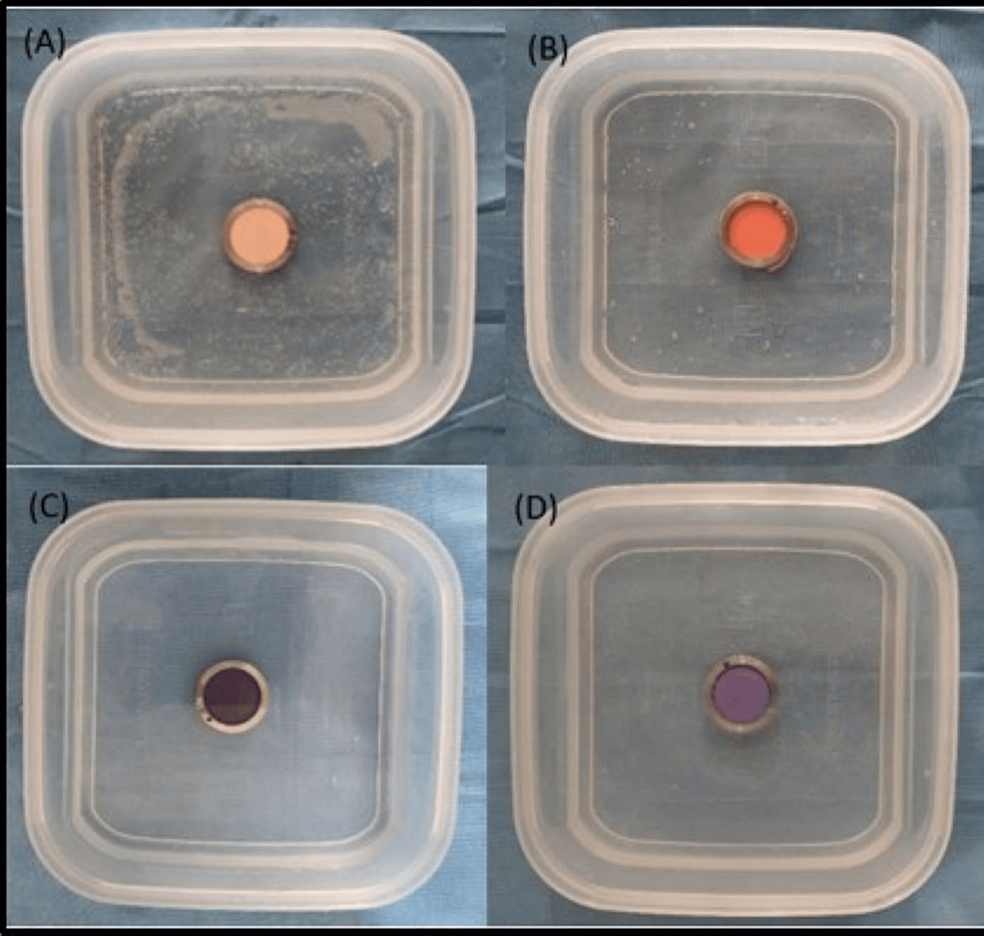
Disinfection of (A) zinc oxide eugenol, (B) alginate, (C) polyether, and (D) addition silicone impressions with 0.2% peracetic acid

**Figure 3:**
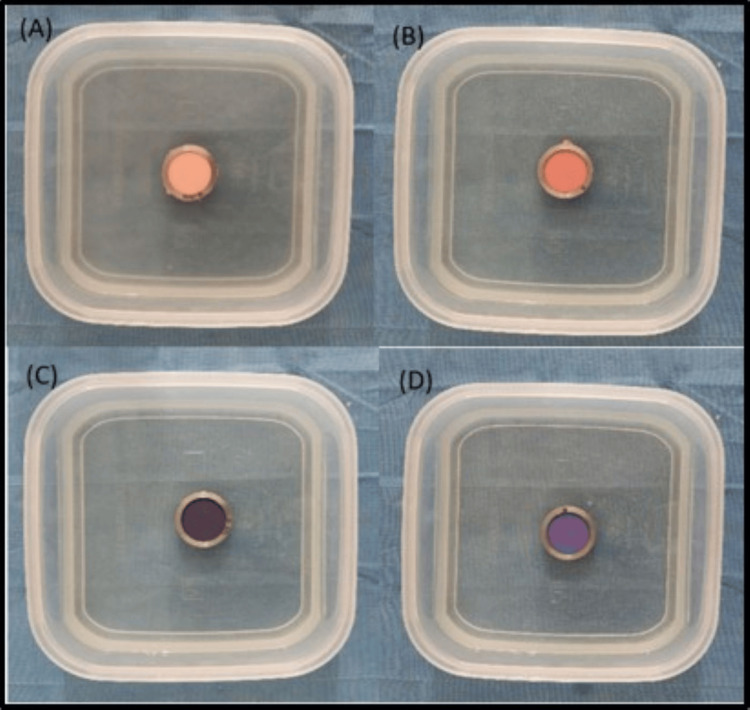
Disinfection of (A) zinc oxide eugenol, (B) alginate, (C) polyether, and (D) addition silicone impressions with a natural polymer of glucosamine

**Figure 4:**
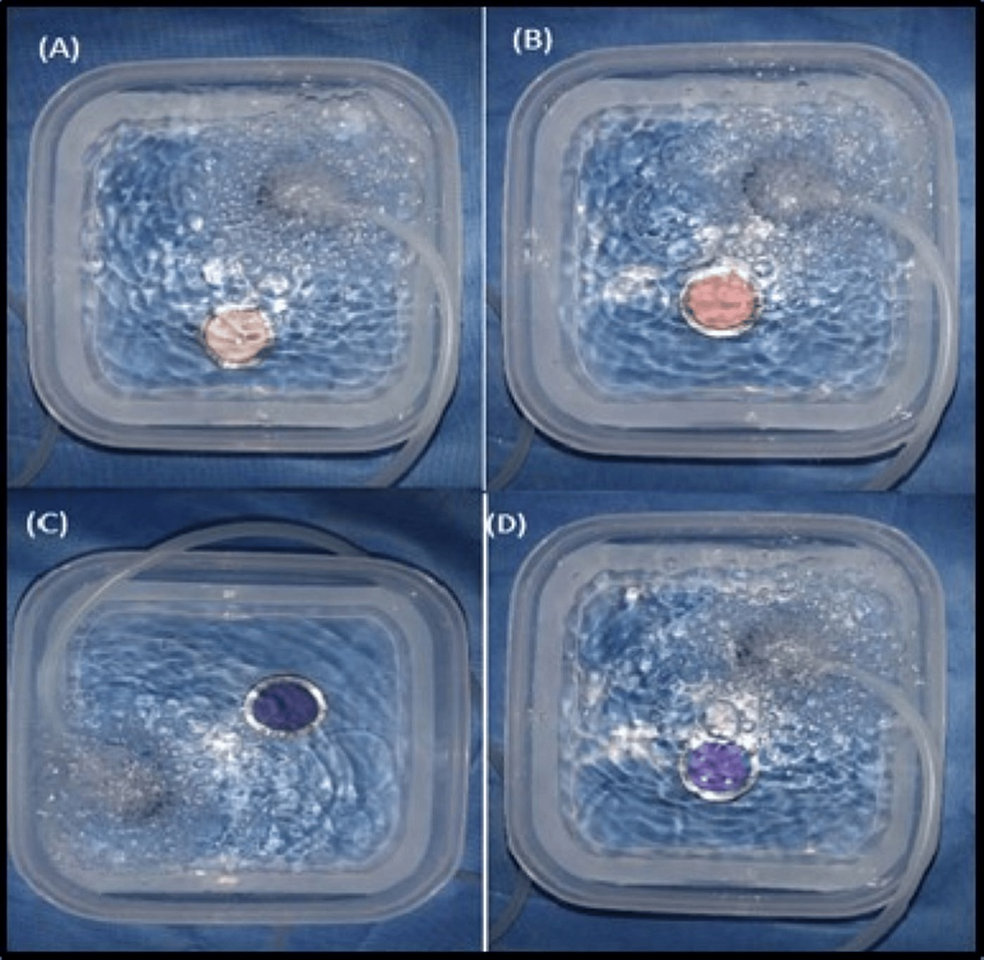
Disinfection of (A) zinc oxide eugenol, (B) alginate, (C) polyether, and (D) addition silicone impressions with ozonated water

The disinfection of the samples was carried out for a duration of 10 minutes by immersion with respect to peracetic acid (Figure 2), a natural polymer of glucosamine (Figure 3), and ozonated water (Figure 4).

**Figure 5:**
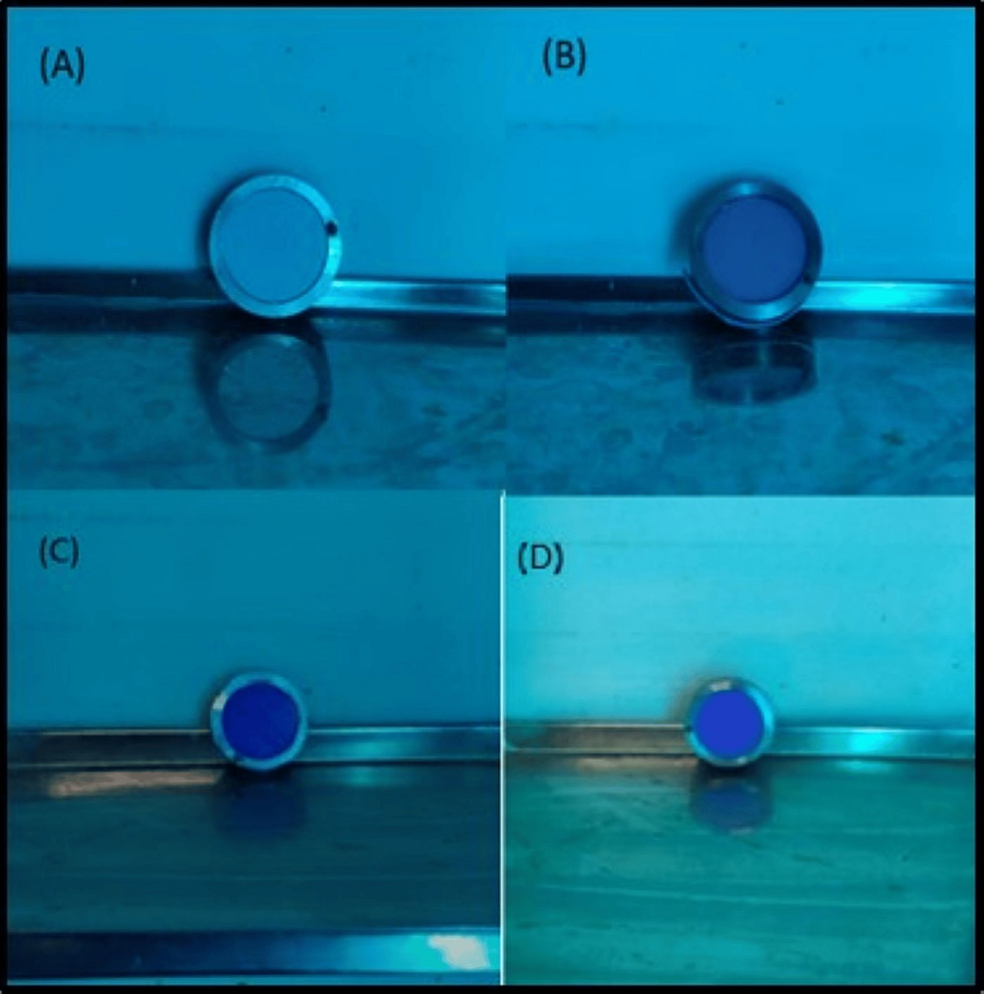
Disinfection of (A) zinc oxide eugenol, (B) alginate, (C) polyether, and (D) addition silicone impressions with UV radiation UV: ultraviolet

A natural polymer of glucosamine disinfecting liquid was diluted to a 2% concentration by mixing 10 mL of the liquid with 490 mL of water before usage. Ozonated water was generated with the help of an ozone generator at a flow rate of 200 mg per hour. The diffuser was placed in a closed clear plastic container containing 1000 mL of water. In case of ultraviolet radiation, the samples were placed in a dental UV chamber that had a UVC lamp installed, for 10 minutes (Figure 5).

**Figure 6:**
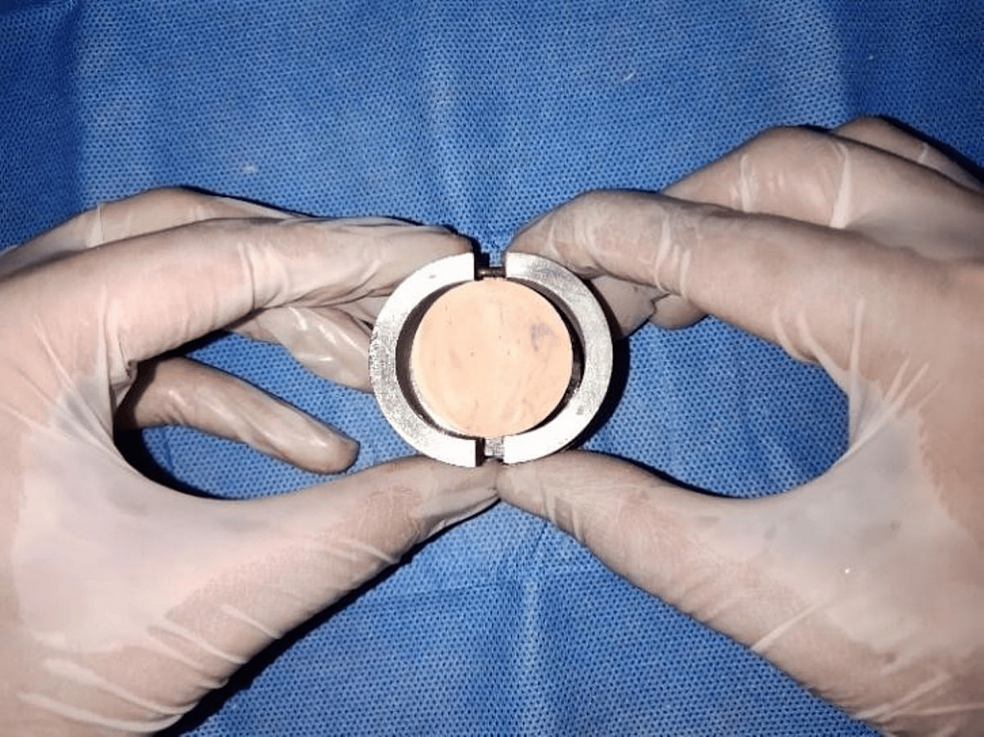
Retrieval of the cast from the split mold

The impressions were poured with type IV gypsum (Kalrock) using the split stainless steel mold. After 15 minutes, casts were retrieved carefully by dismantling the split mold (Figure 6).

**Figure 7:**
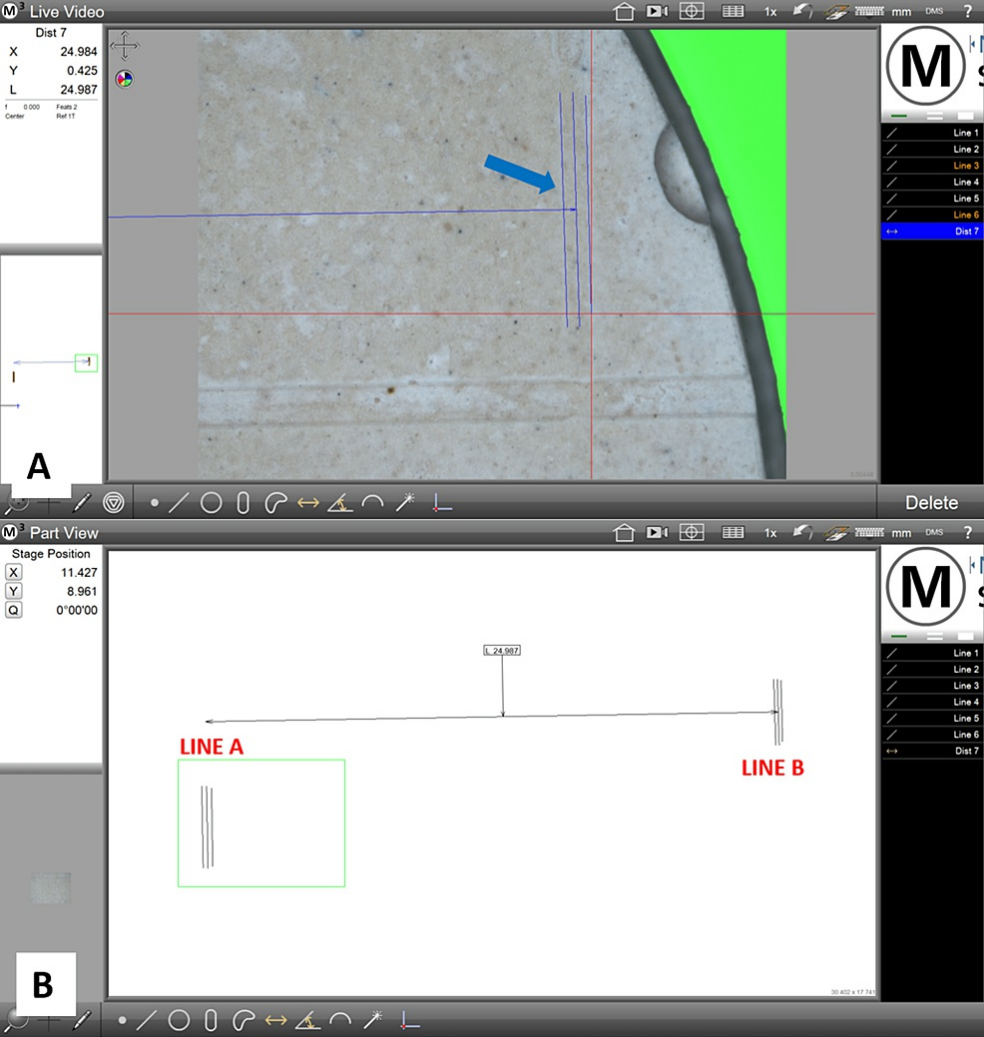
A geometric measuring software (M3 Metrology Readout v3.30.50) being used to calculate the distance between lines A and B (A) Line B (marked with a blue solid line and indicated by a blue arrow) being identified under 50× magnification. (B) The software calculating the distance between lines A and B on a sample cast as 24.987 mm

A video measuring machine (VMM) (SVI-IMG-3D, Sipcon, Dhakola, India) was used to capture the real-time image of the samples. The sample casts were viewed under top light-emitting diode (LED) illumination and a magnification of 50×. With a geometric measuring software (M3 Metrology Readout v3.30.50, MetLogix Inc., Manchester, NH), lines A and B were identified and marked, and the distance between them was measured along the 50 µm line (line X) as specified by ADA specification numbers 18 and 19 (Figure 7). This distance for each sample was noted down as L2. The data was calculated in millimeters accurate to the thousandth place of the value and listed.

The distance between lines A and B on the master model was taken as the reference value, i.e., 25 mm, and was denoted as L1. To calculate the dimensional change observed in each sample, the following formula was applied: dimensional change (in microns)=L1-L2.

From the obtained values of dimensional change per sample, the mean dimensional change was calculated for each subgroup separately.

**Figure 8:**
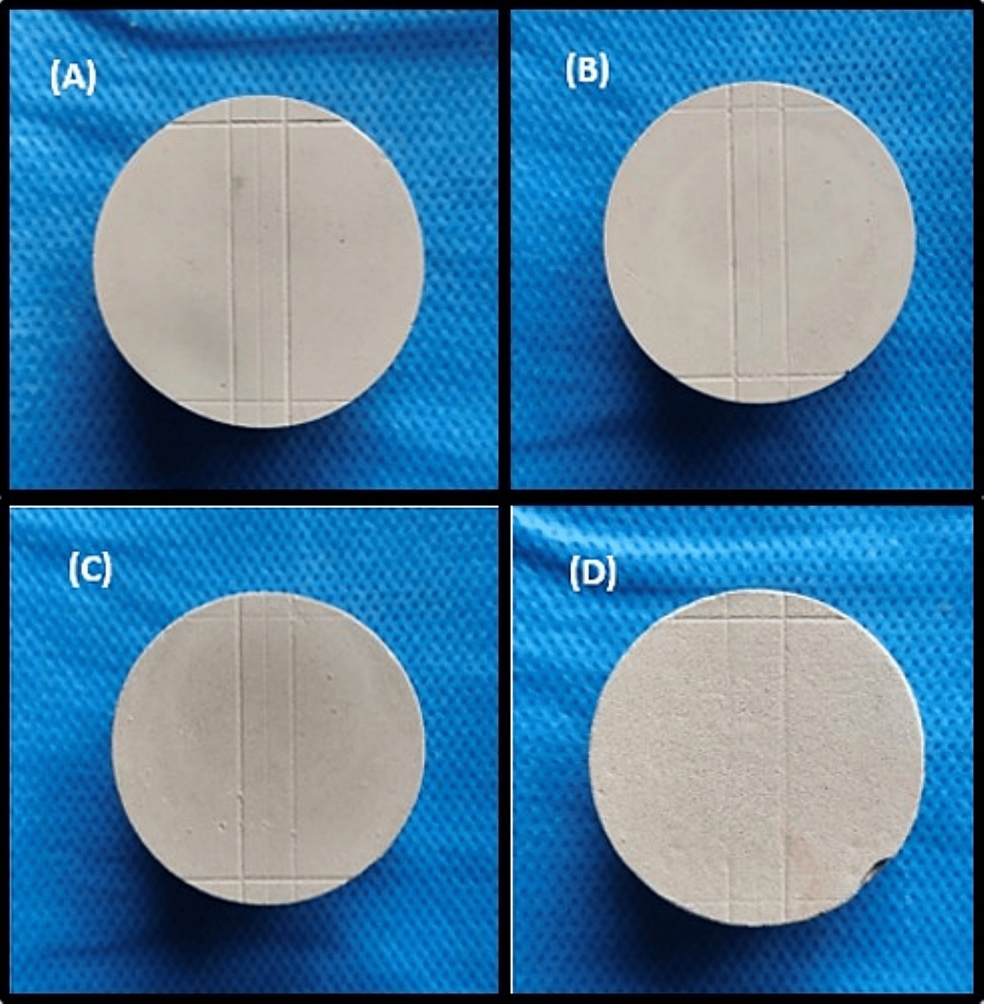
Surface detail reproduction of the samples: (A) Score 1, (B) Score 2, (C) Score 3, and (D) Score 4

Surface detail reproduction was checked with the VMM under a 10× magnification. Line reproduction was considered satisfactory if the 20 µm line (line Y) was continuous between lines A and B. Each sample was then assigned an ordinal score from one to four as the following: Score 1, well-defined, sharp detail and continuous line; Score 2, continuous line but with some loss of sharpness; Score 3, poor detail or loss of continuity of line; and Score 4, marginally or completely not discernable line (Figure 8).

Statistical analysis was carried out using Statistical Package for Social Sciences (SPSS) package 26.0 (IBM SPSS Statistics, Armonk, NY). For dimensional accuracy, inferential statistics was done using a one-way analysis of variance (ANOVA) test to compare the difference between the mean dimensional change values of the subgroups of each impression material. For surface detail reproduction, the chi-square test was used to compare the different subgroups of each impression material separately.

## Results

The lowest mean dimensional change in Group 1 samples was seen in Subgroup 1A (154.1 µm), followed by 1D (176.6 µm) and 1B (214 µm), and the highest mean dimensional change was seen in 1C (249.2 µm). In Group 2 samples, the lowest mean dimensional change was observed in Subgroup 2D (134.9 µm), closely followed by 2B (145.3 µm), 2C (159.3 µm), and 2A (178.6 µm). In Group 3 samples, the lowest mean dimensional change was observed in Subgroup 3C (100.9 µm), followed by 3B (140.6 µm) and 3A (176.3 µm), and the highest mean dimensional change was seen in 3D (216 µm). The lowest mean dimensional change in Group 4 samples was observed in subgroup 4C (113.5 µm), followed by 4D (150.4 µm), 4A (201.7 µm), and 4B (224.7 µm). A one-way ANOVA test was applied, which revealed significant differences (p<0.05) in all subgroups except those of alginate (Group 2) (Table 1).

**Table 1:** One-way ANOVA test for dimensional change Groups (n=40 each): 1, zinc oxide eugenol; 2, alginate; 3, polyether; and 4, addition silicone. Subgroups (n=10 each): A, 0.2% peracetic acid; B, natural polymer of glucosamine; C, UV radiation; and D, ozonated water *Significant difference, p<0.005 #Non-significant difference, p>0.005 ANOVA, analysis of variance; UV, ultraviolet

	Mean dimensional change (µm)		
Subgroups	Mean	Standard deviation	P-value
Subgroup 1A	154.1	28.78	0.001*
Subgroup 1B	214	50.53	
Subgroup 1C	249.2	47.83	
Subgroup 1D	176.6	37.83	
Subgroup 2A	178.6	73.54	0.304#
Subgroup 2B	145.3	53.85	
Subgroup 2C	159.3	41.68	
Subgroup 2D	134.9	36.54	
Subgroup 3A	176.3	33.89	0.001*
Subgroup 3B	140.6	30.01	
Subgroup 3C	100.9	41.92	
Subgroup 3D	216	68.92	
Subgroup 4A	201.7	55.23	0.002*
Subgroup 4B	224.7	53.65	
Subgroup 4C	113.5	46.35	
Subgroup 4D	150.4	89.67	

Table 2 shows the distribution of surface detail reproduction scores for the samples of all subgroups and their comparison using the chi-square test. There was no significant difference (p>0.05) observed in the distribution of SDR scores among the various subgroups of the study.

**Table 2:** Chi-square test for surface detail reproduction Groups (n=40 each): 1, zinc oxide eugenol; 2, alginate; 3, polyether; and 4, addition silicone. Subgroups (n=10 each): A, 0.2% peracetic acid; B, natural polymer of glucosamine; C, UV radiation; and D, ozonated water #Non-significant difference, p>0.005 UV: ultraviolet

Subgroups	Number of samples with surface detail reproduction (SDR) score				P-value
	Score 1	Score 2	Score 3	Score 4	
Subgroup 1A	3	5	2	0	0.127#
Subgroup 1B	4	6	0	0	
Subgroup 1C	5	3	2	0	
Subgroup 1D	1	9	0	0	
Subgroup 2A	0	6	2	2	0.51#
Subgroup 2B	0	8	2	0	
Subgroup 2C	0	8	1	1	
Subgroup 2D	1	6	3	0	
Subgroup 3A	6	3	1	0	0.591#
Subgroup 3B	7	3	0	0	
Subgroup 3C	4	4	2	0	
Subgroup 3D	6	4	0	0	
Subgroup 4A	3	6	1	0	0.481#
Subgroup 4B	6	3	1	0	
Subgroup 4C	7	3	0	0	
Subgroup 4D	4	4	2	0	

## Discussion

The disinfection of impressions before they are poured or handed over to the laboratory personnel is crucial to avoid the cross contamination and transmission of infections, especially viral diseases. There are numerous studies on the disinfection of impressions comparing ADA- to CDC-recommended disinfecting methods for different impression materials. These conventional disinfecting methods are however not proven to be virucidal against more fatal viruses such as SARS-CoV-2, and neither are they compatible with all types of impression materials. With concerns over emerging viral infections, it would be an additional merit if a disinfection method with virucidal properties, as well as compatibility with impression materials, could be used for their disinfection. Be it a diagnostic impression, a secondary impression for a removable prosthesis, or an impression for a fixed prosthesis, the dimensional accuracy of the impression material and the surface detail reproduction of the intraoral anatomy are imperative for a successful treatment. In spite of the inherent properties of impression materials, the surface disinfection of impressions can bring about changes in these physical properties. The present study aimed to test and evaluate the effect of four virucidal disinfection methods, which are not commonly employed for impression disinfection in dentistry, on the dimensional accuracy and surface detail reproduction of different impression materials.

In the present study, zinc oxide eugenol impressions showed the least dimensional change when disinfected with 0.2% peracetic acid whereas the maximum dimensional change was observed when the samples were disinfected using UV radiation. The promising results with 0.2% peracetic acid could be attributed to the oily nature of the impression surface which acted as a protective layer against the disinfectant. Greater dimensional change in samples disinfected with ultraviolet radiation can be explained by the fact that UV radiation being a light source, nonetheless, produces a certain amount of heat that could have affected the dimensional accuracy of zinc oxide eugenol impressions.

The results of the disinfection of alginate impression material showed that the dimensional accuracy of alginate impressions was least affected after disinfection with ozonated water. These results were in accordance with a study conducted by Guiraldo et al. [9]. In this study, both the least and most net dimensional changes were seen when the alginate impressions were disinfected by immersion method. The reason for this difference could be that while ozonated water is a chemically nonerosive material, peracetic acid is a mild acid and could have attacked the surface of the alginate impressions, causing a slightly greater dimensional change as compared to the samples of other subgroups. However, the results of inter-subgroup comparison showed a statistically non-significant difference between alginate impressions disinfected using the various methods.

Polyether impressions showed a statistically significant dimensional change when disinfected by all the disinfecting methods. Ultraviolet radiation was seen to have the least effect on the dimensions of the polyether impressions. This could be attributed to the hydrophilic nature of polyether impression material, which led to greater change in dimensions when disinfected by the other three methods.

Similar to polyether impressions, addition silicone impressions exhibited the least dimensional change when disinfected with ultraviolet radiation. The results of the present study were similar to a study conducted by Nimonkar et al. [10] and Maru et al. [11] who reported little to no significant dimensional changes when polyvinyl siloxane impressions were disinfected with UV radiation for 10 minutes. However, their mean dimensional change was much lesser than that reported in the present study. This could be because, in the present study, many variables could have interfered between the time elapsed from the disinfection procedure and the final measurement of dimensional change such as water-powder ratio, temperature involved during manipulation of gypsum products, setting reaction, and setting expansion.

According to ADA specification numbers 18 and 19, the impression materials used to fabricate precision castings must be able to reproduce fine detail to a level of 20 µm or less. In the present study, most of the zinc oxide eugenol impressions received a satisfactory score of 2 (continuous line but with some loss of sharpness). A study conducted by Hummudi [12] also reported a loss of surface detail and an increase in surface roughness when zinc oxide eugenol impressions were disinfected with acetic acid-based disinfectants. Alginate impressions displayed a loss of surface detail reproduction to a certain extent in all the samples after disinfection. Only one sample that was disinfected by ozonated water received a score of 1. This could be due to the imbibition and syneresis properties of alginate impression materials. On the other hand, both polyether and addition silicone impressions showed an above-average exhibition of surface detail reproduction after disinfection, similar to a study by Abinaya et al. [13].

However, the results for all four impression materials showed no statistically significant difference after disinfection with the four disinfecting methods. This was in accordance with studies conducted by Shambhu and Gujjari [14], Guiraldo et al. [9], and Rabeeba et al. [15].

Peracetic acid is a high-level disinfectant, which is biodegradable and nontoxic. A peracetic acid of 1500 ppm (equivalent to 0.25%) is known to be virucidal. Glucosamine, mainly obtained from chitin, is a biopolymer that is known to be active against various animal viruses. Its use as an impression disinfectant was studied by Jha et al. [16] who reported affirmative results when used for the disinfection of alginate impressions.

Ultraviolet light radiation, which has been introduced in recent decades, is lethal to bacteria, bacterial spores, viruses, mold, mold spores, yeast, and algae in 200-280 nm wavelength [17]. Thus, impressions can be disinfected in a dental UV chamber where a UV light of wavelength 254 nm is emitted so that the impression is simultaneously exposed to UV radiation from different directions. Aeran et al. [17] showed the complete disinfection of alginate, polyether, and addition silicone impressions after a 10-minute exposure to UV radiation.

Ozone disinfection is a new method, which needs no consumables, is time-saving, and requires limited space in the dental office. This minimizes liquid waste generation resulting in superior environmental protection [18,19]. Savabi et al. [20] showed, with the help of their study, that a 10-minute immersion of irreversible hydrocolloid impression material in ozonated water can reduce the number of microorganisms.

A limitation of this study was that the dynamic intraoral conditions were not simulated. Various factors such as the presence of saliva, blood, and pus can compromise the efficiency of disinfectants in reducing the microbial load. These residues can act as a barrier to the actual effects of the disinfectant on the dimensional accuracy and surface detail reproduction of the impressions. Moreover, the present study utilized only a specific shape of samples as dictated by the design of the master model. The retrieval of samples from the master model was not hindered by any obstacles, for example, undercuts as encountered in a patient’s mouth. Hence, distortion due to impression recovery was obliterated. Also, the dimensional change was recorded by taking measurements in a single plane only.

There are very few studies that have evaluated the effect of virucidal disinfectants on the dimensional accuracy and surface detail reproduction of zinc oxide eugenol impression. Further investigations are required to test the efficiency of these emerging virucidal disinfectants with the help of clinical studies.

## Conclusions

The results of the effect of virucidal disinfectants on dimensional accuracy were significant, and based on these results, recommendations can be safely made. Zinc oxide eugenol impressions can be disinfected by a 10-minute immersion in 0.2% peracetic acid. Based on the results, ozonated water showed the least effect on the dimensional accuracy of alginate impressions and hence could be recommended as a virucidal disinfectant. Both polyether and addition silicone impressions can be disinfected with a 10-minute exposure to ultraviolet radiation without causing a significant change in dimensional accuracy. None of the virucidal disinfectants significantly affected the surface details of these impression materials and could be used appropriately.

From the observation of results, it can be concluded that the tested virucidal disinfectants, which were peracetic acid, a natural polymer of glucosamine, UV radiation, and ozonated water, can be better alternatives to the conventionally used disinfectants in terms of biocompatibility, the ease of use, and economics. Also, the effect of these disinfectants on the dimensional accuracy and surface detail reproduction were within the maximum limits dictated by the ADA standards.
